# Refeeding Syndrome in Emergency Department Boarders: How COVID-19 Can Reshape Patient Care in the Emergency Department

**DOI:** 10.7759/cureus.23874

**Published:** 2022-04-06

**Authors:** Patrick Meloy, Lauren Howell, Emily Armstrong, Elaine Bromberek

**Affiliations:** 1 Emergency Medicine, Emory University School of Medicine, Atlanta, USA; 2 Pharmacy, Emory University School of Medicine, Atlanta, USA

**Keywords:** covid-19 pneumonia, electrolyte derangement, emergency department boarding, covid-19, refeeding syndrome, critical care, emergency medicine

## Abstract

Emergency departments (EDs) in the United States are the primary drivers of hospital admissions. As the nation continues to experience unrestrained spread of the severe acute respiratory syndrome coronavirus 2, causing coronavirus disease 2019 (COVID-19), EDs, hospitals, and testing centers are overwhelmed with patients. The consequence of “boarding” admitted patients in EDs leads not only to longer ED wait times for all patients but also delays the medical practice of intensivists and internists while patients await an inpatient bed. Here, we describe the case of an ED boarder with severe COVID-19 who developed refeeding syndrome while boarding in the ED, ultimately requiring in-depth electrolyte and renal management by the ED team before intensive care unit admission.

## Introduction

In 2018, the number of emergency department (ED) visits topped 143 million in the United States [1]. In the same year, 70% of hospital admissions funneled through the ED [2]. Since the onset of the severe acute respiratory syndrome coronavirus 2 (SARS-CoV-2) pandemic, emergency physicians (EPs) and other members of the emergency medicine (EM) team have been on the frontlines of the pandemic, learning to manage patients with coronavirus disease 2019 (COVID-19) and its associated sequelae along with caring for patients with historically common emergencies. As the pandemic continues to progress, ED visits and hospitalizations continue to follow suit, increasing in number and severity. EPs continue to be not only the first healthcare providers for COVID-19 patients but are also providing extended care as hospitals are pushed above their normal operating capacity [3]. As SARS-CoV-2 continues to undergo genetic modifications and variants continue to be identified, viral transmission will continue to occur and lead to continued pressure and burden on EDs and already struggling health systems [3,4]. In conjunction with ongoing elevations in COVID-19 cases and hospitalizations, the United States is experiencing an unprecedented nursing crisis [5]. The lack of available nursing staff has decreased overall nursing hours, which directly contributes to the extensive boarding time of patients in the ED [3]. A lack of movement throughout the hospital ultimately burdens EPs, the EM healthcare team, and patients who are unable to be seen in the ED.

## Case presentation

A 62-year-old-female with a medical history of stroke with right-sided residual deficits and hypertension presented to the ED with altered mental status. According to her family members, at baseline, she was bedridden, with the ability to feed herself and verbally communicate clearly. She was last seen in her normal condition approximately three days before arrival by a family member. The patient’s primary caretaker, her daughter, had become ill with COVID-19 preventing her from taking care of the patient. For approximately three days, the alternate caregiver, her niece, did not communicate changes in the patient’s mental status to other family members. Thus, it is unclear if she was receiving her routine medications, being fed, or what care was being provided. Upon seeing the patient, the extended family activated the local EMS. Upon EMS arrival, the patient was found to be profoundly hypoglycemic, with a blood glucose of 30 mg/dL per fingerstick. There was no history of diabetes, anti-hyperglycemic medications, or insulin use. She was given oral glucose with only minimal improvement in her blood sugar, remained altered, and was subsequently transported to the ED for management of unexplained hypoglycemia. Initial vitals in the ED showed the patient was hypertensive (156/97 mmHg), tachycardiac (117 beats per minute), and afebrile (36.7°C). She was notably tachypneic (22 breaths per min) and moderately hypoxic (90% oxygen saturation on room air).

The patient was seen by an EP immediately upon arrival; intravenous (IV) catheter access was established and she was given 25 g of IV glucose (D50). Her blood sugar increased to >500 mg/dL, but her mental status did not change. The patient was volume resuscitated with 1 L of lactated ringers and underwent a broad workup, including a comprehensive metabolic panel, complete blood count, lactic acid, venous blood gas, COVID-19 swab, blood and urine cultures, chest radiograph (CXR), non-contrast computed tomography of the head (CT-head), and electrocardiogram (EKG). Remarkable findings upon ED presentation included elevated glucose, sodium, lactic acid, and anion gap, with decreased potassium (Table 1).

**Table 1:** Lab values on presentation and over time in the emergency department. BUN: blood urea nitrogen; pCO_2_: partial pressure of carbon dioxide; pO_2_: partial pressure of oxygen; WBC: white blood cell count

	Initial	12 hours after presentation	16 hours after presentation	24 hours after presentation	Reference range
Sodium	150	142	146	145	136–145 mmol/L
Potassium	3.2	2.8	3.3	5.9	3.5–5.1 mmol/L
Carbon dioxide	21	19	18	22	23–29 mmol/L
Anion gap	24	24	25	13	2–11 mmol/L
Glucose	495	389	217	215	70–105 mg/dL
BUN	31	29	27	25	7–31 mg/dL
Creatinine	1.11	0.89	0.83	0.61	0.6–1.2 mg/dL
Phosphorus	Not obtained	Not obtained	<1.0	6.2	2.5–5.0 mg/dL
Magnesium	Not obtained	Not obtained	1.7	2.6	1.9–2.7 mg/dL
Lactic acid	7.8	8.6	10.9	4.5	0.5–2.2 mmol/L
pH	7.36	7.43	Not obtained	7.38	7.32–7.43
pCO_2_	37	20	Not obtained	41	41–51 mmHg
pO_2_	35	68	Not obtained	112	35–42 mmHg
Bicarbonate	20.4	13.1	Not obtained	23.4	21–28 mmol/L
WBC	11.9	16.4	14.8	13.8	4–10 × 10^3^/µL
Hemoglobin	15.8	15.8	16.0	12.6	11.4–14.4 g/dL
Hematocrit	47.0	46.2	46.5	36.6	33.3–41.4%
Platelets	197	144	141	103	150–400 × 10^3^/µL

**Figure 1:**
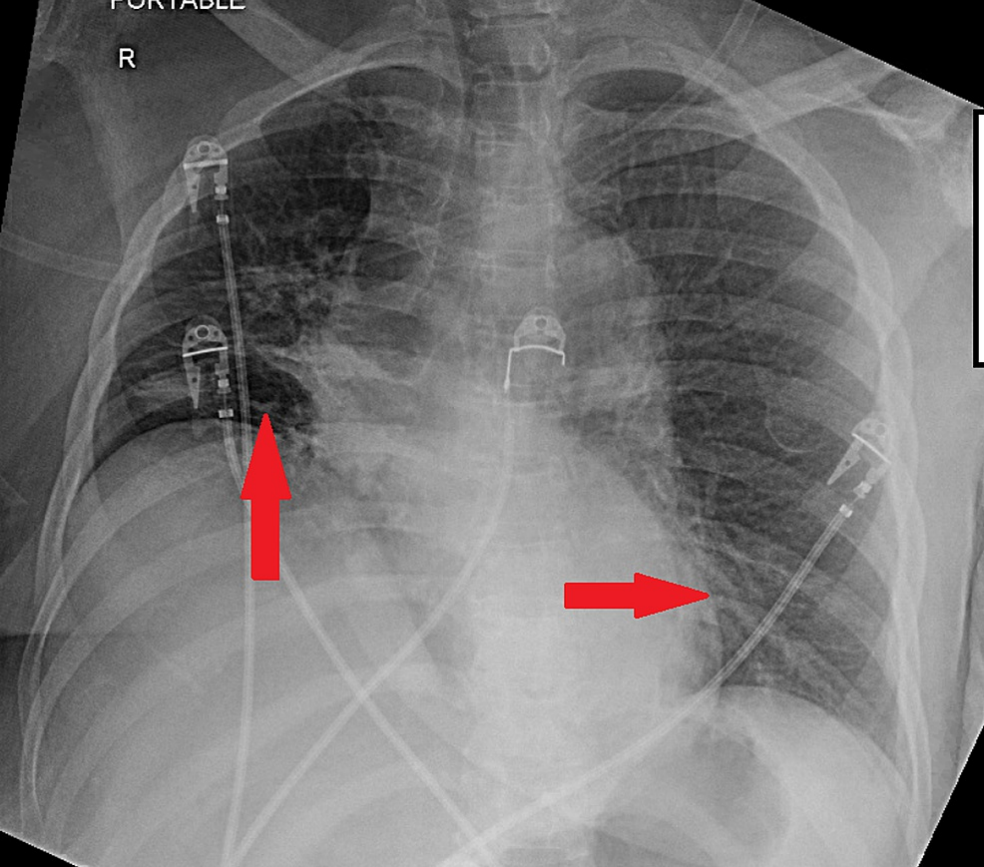
Portable chest radiograph indicating right perihilar, right lower lobe, and left lower lung airspace opacities (red arrows), consistent with multi-segment or viral pneumonia.

CT-head showed chronic microvascular changes with no acute abnormalities. The CXR (Figure 1), though severely rotated, indicated bilateral lower lobe airspace opacities which were consistent with multi-segment pneumonia or viral pneumonia. Further, 12-lead EKG was interpreted as sinus tachycardia with a rate of 110 beats per minute, right axis deviation, ST-segment depression in leads I, II, III, aVF, V5, and V6, with no corresponding ST-segment elevation; no prior EKGs were available for comparison. Rapid COVID-19 testing returned as positive.

After review of the patient’s initial laboratory results and imaging, the patient was administered a second liter of lactated ringers and started on ceftriaxone, 1 g every 24 hours, and intravenous doxycycline, 100 mg every 12 hours, for pneumonia. Due to her ongoing work of breathing, bilevel positive airway pressure (BiPAP) was initiated with the following settings: FiO_2_ 35%, expiratory positive airway pressure (EPAP) 6 cmH_2_O, inspiratory positive airway pressure (IPAP) 12 cmH_2_O. She was able to shake her head to indicate she did not want intubation. A repeat venous blood gas was obtained after two hours which indicated worsening lactic acidosis and recurrent hypoglycemia. Moreover, phosphorus and magnesium were profoundly low (Table 1). At this time, she was started on a dextrose 10% infusion at 75 mL/hour to manage her recurrent hypoglycemia, and a request was made for intensive care unit (ICU) bed placement. Unfortunately, there were no available beds, and the patient was boarded in the ED for ongoing care.

Given her complex metabolic, infectious, and respiratory needs, the ED pharmacist was consulted. In light of the patient’s clinical picture, antibiotics were broadened to vancomycin and cefepime, and COVID-19 treatment with dexamethasone and remdesivir was initiated. Additionally, treatment for the possible refeeding syndrome was initiated. This included an insulin infusion at 0.1 units/kg, aggressive electrolyte replacement with potassium phosphate 30 mmol/L intravenous piggyback (IVPB), magnesium 4 g IVPB, and 0.9% NaCl with 40 mEq KCl (40 mEq/L) at 150 mL/hour. The patient remained in the ED under the EM team’s care for over 24 hours. During this time, she had significant improvement in her electrolytes, but, unfortunately, due to her ongoing respiratory and infectious symptoms, she still required admission to the medical ICU.

The patient had an extensive hospital stay. She was treated for aspiration pneumonia and found to have multiple new strokes. Her dysphagia worsened and her aphasia continued despite appropriate resolution of metabolic encephalopathy. Once transferred to the floor, the patient and her family requested palliative care and hospice consult instead of the recommendation of a subacute rehab facility. On hospital day 42, the patient was released to home hospice, with her daughter as her primary caretaker.

## Discussion

While the COVID-19 pandemic is recognized as a global health crisis, the United States in particular has borne the brunt of the effects of the ongoing number of cases [6]. As one of the global leaders in cumulative COVID-19 deaths, the US healthcare system has been under tremendous pressure during the pandemic, with US EDs at the forefront [6]. While the pandemic has not changed the pressure on healthcare systems to efficiently move patients through hospital admission, current circumstances in the United States have led to the increasingly common practice of boarding patients in the ED, which leads directly to longer stays, more inefficient care, and increased morbidity and mortality [7]. As inpatients require longer ED stays, EPs are required to focus on inpatient medicine, which adds another level of complexity to distract them from the routine care of undifferentiated ED patients [7]. EPs are specialized in emergency management and stabilization but are not experts in the ongoing, multi-day management of patients. This further divides attention and prohibits the treating physician from lengthy, in-depth case review with undivided attention [8]. This case is an example of an ICU-level patient, managed in the ED, who required aggressive interventions for her initially unrecognized refeeding syndrome.

Refeeding syndrome is best defined as a severe electrolyte and fluid shift in malnourished patients who are undergoing parenteral, enteral, or oral refeeding [9]. Patients experience severe alterations in fluid balance and can develop life-threatening hypoglycemia, hypomagnesemia, and hypophosphatemia [9]. Refeeding syndrome was first described in Japanese prisoners of war around World War II [10]. It is now recognized in patients who have undergone prolonged fasting, elderly and chronically malnourished patients, and anyone who has had an inadequate diet over a period of seven days [9]. In this case, the patient had been unintentionally neglected while her family struggled with their own COVID-19 infections, which was ultimately passed onto the patient and worsened her overall presentation.

During the period of starvation, a combination of gluconeogenesis and glycogenolysis occurs to provide the body with adequate glucose levels [9,11]. Protein and lipids are broken down initially. Adipose tissue is broken down into fatty acids, and muscle tissue is broken down into amino acids. When glucose is not available, the body uses ketone bodies and free fatty acids as its primary energy source [9,11]. As the patient is fed, whether orally or parenterally, the shift to glycolysis occurs, and insulin is released to facilitate this change. This rapid increase in blood insulin levels leads to cellular uptake of fluids and electrolytes and initiates protein creation, leading to the pathophysiology of the refeeding syndrome [12]. This critically low level of electrolytes puts patients at high risk for cardiac dysrhythmias, left ventricular dysfunction due to cardiac atrophy, hypotension, and Wernicke’s encephalopathy [13].

In this case, the patient was severely malnourished and hypoglycemic and was rapidly administered multiple loading doses of IV glucose. This initial bolus dosing led to confounding information on her initial lab results, creating a concern for possible diabetic ketoacidosis given the elevated anion gap, elevated ketones in her urine, and markedly elevated lactic acid. The patient did not have an initial phosphorus level tested, but this was likely already depleted secondary to the level of phosphorus use in the production of adenosine triphosphate. As insulin levels rapidly increased to counteract the concurrent rapid administration of glucose, the inevitable consequence was a significant intracellular shift of blood electrolytes and critically low phosphorus of <1.0 mg/dL.

Appropriate management of refeeding syndrome is controversial due to the lack of randomized controlled trials on the subject [14]. Recommendations from the American Society for Parenteral and Enteral Nutrition include avoiding refeeding syndrome by checking electrolytes before feeding and facilitating the replacement of electrolytes, as needed, to maintain appropriate homeostatic activities [15]. When time is not critically short, patients should have electrolytes managed before an increase in their caloric intake, although this is not typically available to EPs in most scenarios. When refeeding syndrome is diagnosed, the electrolyte replacement should take place over 48-72 hours, and the patient should have a low-calorie diet introduced until the first 72 hours have passed [15].

Our patient’s condition was subsequently exacerbated by the initiation of an insulin drip, which led to worsening electrolyte and fluid shift intracellularly, requiring management with a continuous IV glucose infusion and continuous electrolyte replacement. She did not receive IV thiamine. Although the patient did not suffer from cardiac dysrhythmias because of her profound electrolyte abnormalities, the combination of severe hypokalemia, hypophosphatemia, and hypomagnesemia has dangerous potential. Though her initial ED management was not optimized based on all the available evidence, the patient was resuscitated appropriately with periodic electrolyte supplementation in the ICU and ultimately had a stable resolution of her refeeding syndrome over the next 72 hours.

## Conclusions

Our patient presented to the ED with clinical signs and symptoms of severe malnutrition, which went unrecognized in the preliminary management due to her ongoing altered mental status and concurrent infection with COVID-19. The patient was initially treated with an IV insulin infusion, which likely exacerbated her refeeding syndrome, potentially leading to dangerous cardiac dysrhythmias. This case reiterates that EPs need to be vigilant in their care of elderly and chronically malnourished patients in the ED, which includes chronic alcoholics, homeless, and nursing home patients. The over-capacity in hospitals combined with historic shortages in nurse and clinician staffing has led to an increase in the practice of boarding inpatients in the ED before bed availability.
